# Cadmium SAD phasing at CuKα wavelength

**DOI:** 10.12688/f1000research.17694.1

**Published:** 2019-01-21

**Authors:** Igor E. Eliseev, Anna N. Yudenko, Valeria M. Ukrainskaya, Oleg B. Chakchir

**Affiliations:** 1 St. Petersburg National Research Academic University RAS, St. Petersburg, 194021, Russian Federation

**Keywords:** Protein crystallography, experimental phasing, single-wavelength anomalous diffraction, cadmium ions, Cd-SAD

## Abstract

Single-wavelength anomalous diffraction (SAD) is the most common method for *de novo* elucidation of macromolecular structures by X-ray crystallography. It requires an anomalous scatterer in a crystal to calculate phases. A recent study by Panneerselvam *et al*. emphasized the utility of cadmium ions for SAD phasing at the standard synchrotron wavelength of 1 Å. Here we show that cadmium is also useful for phasing of crystals collected in-house with CuKα radiation. Using a crystal of single-domain antibody as an experimental model, we demonstrate how cadmium SAD can be conveniently employed to solve a CuKα dataset. We then discuss the factors which make this method generally applicable.

## Introduction

Elucidation of atomic structures of macromolecules by X-ray crystallography requires knowledge of the phases of measured reflections. Nowadays this phase problem is most often solved by molecular replacement (MR), a computational technique which utilizes the known structure of a homologous molecule to estimate phases. However, in the case of *de novo* structure elucidation when an appropriate homologous structure is unavailable, phases should be determined experimentally. This is predominantly achieved by analyzing anomalous scattering produced either by atoms naturally occurring in the molecule, or intentionally introduced into crystal during growth or soaking. The two phasing methods exploiting the anomalous scattering, multiwavelength anomalous diffraction (MAD) and single-wavelength anomalous diffraction (SAD), were reviewed by Hendrickson ^1^. Synchrotron radiation with tunable wavelength allows achieving the absorption edges of all elements with Z≥20 to maximize anomalous signal, thus making these methods remarkably versatile.

On the contrary, the choice of anomalous scatterer is minimal when data are to be collected in-house using a laboratory X-ray generator, most often equipped with a copper anode (λ=1.5418 Å, CuKα). Indeed, in some cases, even weak anomalous signal of sulfur ( *f′′*=0.56e ^-^ at CuKα) can be used for phasing, as demonstrated in pioneering SAD work on crambin ^2^. Similarly, zinc ( *f′′*=0.68e ^-^ at CuKα) was proposed to be useful for in-house SAD experiments ^3^. Perhaps the most impressive result came from the structural genomics project, where iodine ion soaks were systematically used for *de novo* SAD phasing of datasets collected with CuKα radiation ^4^. Iodine has a strong anomalous scattering ( *f′′*=6.9e ^-^ at CuKα), high solubility, and binds multiple hydrophobic sites or positively charged residues on protein surface. Iodine SAD appeared remarkably efficient for phasing the crystals of membrane proteins which possess patches of positively charged residues at the hydrophobic-hydrophilic interface, providing many binding sites for anions ^5^.

Another attractive opportunity is to use cadmium ions, which have a great anomalous signal ( *f′′*=4.7e ^-^ at CuKα) comparable to that of iodine, promote crystal growth ^6^, and can substitute other divalent cations in metal-binding proteins. Despite all these advantages and its use in the very early SAD works ^7^, Cd is rarely used in the phasing of protein crystals. Recently, a paper emphasizing the utility of cadmium ions for experimental phasing at the standard synchrotron wavelength of 1 Å was published ^8^. In this short research note, we show how Cd-SAD can also be conveniently used for phasing datasets collected using CuKα radiation.

## Methods

As an experimental model for in-house cadmium SAD, we used a crystal of an anti-ErbB3 single-domain antibody BCD090-M2, which we recently studied ^9^. The details of protein purification, characterization, and structural analysis are given in the paper ^9^. Briefly, the protein was expressed in *E. coli SHuffle* cells as a SUMO fusion, purified by immobilized metal affinity chromatography, cleaved by TEV protease, and then polished by an additional step of high-resolution cation-exchange chromatography. The antibody was crystallized by hanging-drop vapor diffusion in two different forms: in a space group C2 without divalent cations (PDB accession number: 6EZW) and in P1 with two cadmium ions per unit cell (PDB accession number: 6F0D) ^9^. Crystals of both types diffracted below 2 Å. The data were collected on a Kappa Apex II diffractometer (Bruker AXS) using CuKα radiation generated by a IμS microfocus X-ray tube. Both structures were solved by molecular replacement in Phenix software suite v. 1.11 ^10^. The dataset with cadmium (6F0D) with unmerged Friedel pairs was used for SAD analysis. For experimental phasing, we used a standard protocol employing *SHELXC/D/E* programs ^11^ through *HKL2MAP* v. 0.4 graphical interface ^12^. Data were processed with *SHELXC* v. 2016/1, anomalous substructure was solved by *SHELXD* v. 2013/2 and phasing and density modification were done by *SHELXE* v. 2018/2. The automatic model building and refinement were done in Phenix v. 1.14 ^10^, and manual refinement was done in *Coot* v. 0.8.9.1 ^13^. Figures were prepared with *PyMOL*.

## Results and discussion

**Figure 1.**
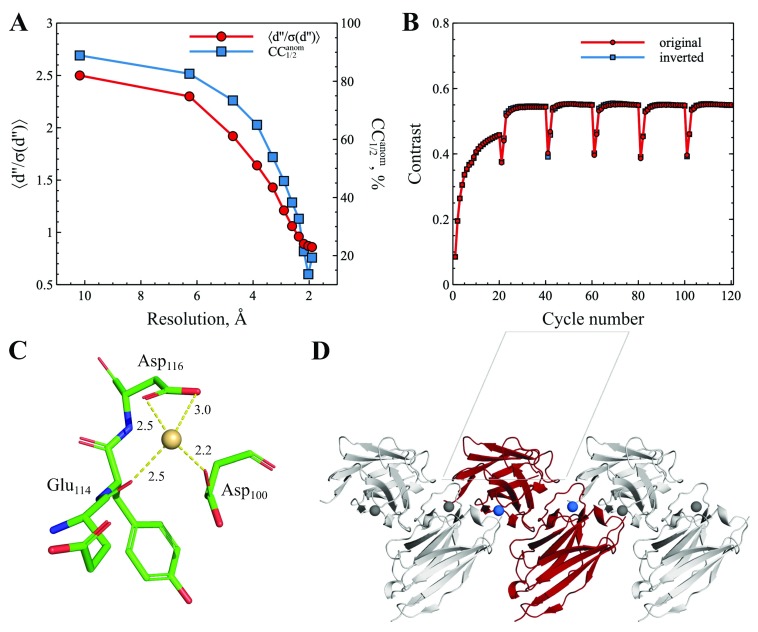
Cadmium SAD phasing of the dataset collected at CuKα wavelength. The crystal of the single-domain antibody BCD090-M2 with cadmium ions was used as an experimental model for in-house Cd-SAD. ( **A**) Strength of the anomalous signal represented by ⟨d′′/σ(d′′)⟩ and CC _1/2_ as a function of resolution. ( **B**) Electron density modification in SHELXE as monitored by an increase in map contrast; solutions with original and inverted anomalous substructure give indistinguishable contrast due to centrosymmetry. ( **C**) Cadmium ion binding site. ( **D**) Schematic representation of the crystal unit cell.

The phasing of protein crystals by SAD starts from finding the positions of an anomalous substructure, which is usually done by direct methods. First, the dataset was processed with *SHELXC*, and the statistical analysis of the anomalous signal is shown in Figure 1A and Table 1. The use of kappa goniometer for data collection allowed achieving high completeness (96.4%) and multiplicity (5.9) of anomalous pair measurements. The signal-to-noise ratio defined as ⟨d′′/σ(d′′)⟩ and the correlation coefficient CC _1/2 _indicate that useful anomalous signal is present almost in the whole resolution range. For further substructure solution, we implied a rather conservative high-resolution cut-off of 2.4 Å corresponding to CC _1/2_ (anom.) ~ 0.3.

**Table 1.** Cadmium SAD phasing and model building results. Values in parentheses are for the highest resolution shell.

Parameter	Value
*Dataset statistics (6F0D)*	
Space group	P1
Unit cell: a b c (Å) α β γ (◦)	35.77 41.53 46.49 89.99 67.92 76.06
Resolution range, Å	32.34–1.90 (1.94–1.90)
Reflections: total / unique	246903 (6236) / 18859 (1265)
Completeness (all), %	100.0 (100.0)
Completeness (anom.), %	96.4 (89.9)
Multiplicity	13.1 (4.9)
Multiplicity (anom.)	5.9 (2.4)
Mean I/σ(I)	14.9 (2.2)
*SHELXD*	
Resolution range, Å	32.34–2.4
CFOM	55.61
CC _all_/CC _weak_	32.40 / 23.21
No. of sites	2/2
*SHELXE*	
No. of residues built	222 / 256 (87%)
CC	43.57
*phenix.autobuild*	
No. of residues built	245 / 256 (96%)
CC	0.80
*Refinement*	
No. of residues built	256/256 (100%)
CC	0.90
R _work_ / R _free_, %	17.8 / 21.0

CFOM, combined figure of merit; CC, correlation coefficient.

 The anomalous substructure was immediately solved by *SHELXD* as judged by high correlation coefficients (combined figure of merit = 55.6%), high occupancies of the two cadmium sites (1.00, 0.99), and the rapid drop in occupancy of the next site (0.17). The positions of Cd ions corresponded to the largest off-origin peak of the anomalous Patterson function at (0.58, 0.02, 0.03). The solution was used in *SHELXE* for phasing, electron density modification, and chain tracing. This yielded electron density maps with high contrast, and the solutions for original and inverted substructure were indistinguishable due to centrosymmetry ( Figure 1B). As discussed previously ^14^, centrosymmetric anomalous sites in SAD can impede interpretation of electron density maps, because the resulting map is a superposition of the true electron density with its negative mirror-image. However, in our case the major portion of the protein chain (87%) was traced after density modification. This incomplete model was further improved in *phenix.autobuild*, and then refined manually in *Coot* and *phenix.refine* giving final R _work_/R _free_ of 17.8/21.0%.

 In this particular case, structure determination by in-house Cd-SAD was almost as straightforward as an automated molecular replacement. The causes of this simplicity were the relatively small protein size, high completeness and multiplicity of the anomalous data, and the small number of high-occupancy cadmium sites. Furthermore, the recent theoretical study gives the following simple dependency for expected anomalous signal ⟨S _ano_⟩ ~ (N _refl_/n _sites_) ^1/2^, where N _refl_ is the number of independent reflections and n _sites_ is the number of anomalous scatterers ^15^. Our case with maximum N _refl_ due to the lowest symmetry (P1) and only 2 anomalous sites appears virtually optimal for SAD. The high metal-binding affinity of cadmium sites was achieved through coordination with carbonyl oxygen of Glu _114_, and carboxylic groups of Asp _100_ and Asp _116_ ( Figure 1C). By bridging these residues to the N-terminal Gly residue of the neighboring molecule, cadmium ions effectively defined crystal contacts ( Figure 1D). Data associated with this study are available on OSF ^16^.

## Conclusion

In conclusion, we suggest that cadmium SAD can be generally applied for the phasing of protein crystals collected in-house using CuKα radiation. We see the following advantages of this approach: (1) cadmium has a great anomalous signal ( *f′′*=4.7e ^-^ at CuKα); (2) cadmium ions frequently promote crystal growth and can substitute other divalent cations; (3) cadmium binding sites are complementary to that of iodine, another strong anomalous scatterer, and therefore Cd-SAD can be useful in cases where I-SAD does not work.

## Data availability

Data for this study, including unmerged experimental intensities, structure factors and final atomic coordinates after refinement, are available on OSF. DOI: https://doi.org/10.17605/OSF.IO/KYH6D ^16^.

Data are available under the terms of the Creative Commons Zero “No rights reserved” data waiver (CC0 1.0 Public domain dedication).
