# Supplementary material for: Cadmium SAD phasing at CuKα wavelength
Source: F1000Res. 2019 Jan 21;8:84. [Version 1] doi: 10.12688/f1000research.17694.1 (PMC6446493; doi:10.12688/f1000research.17694.1)
Supplement: Peer review history

# Peer review: Reviewer response for version 1

**DOI:** 10.5256/f1000research.19346.r45676

**Published:** 2019-04-01

**Authors:** Igor Melnikov

**Affiliations:** 1 European Synchrotron Radiation Facility (ESRF), Grenoble, France

In this experiment report the authors present their work on derivatising crystals of a single-domain antibody protein with cadmium ions and subsequent solving the structure via SAD. They demonstrate that home-lab X-ray crystallographic equipment is capable of solving crystal structures via SAD at CuKalpha X-ray energy. The authors then conclude by discussing the result and suggesting the method for use in home-lab source diffraction experiments. To sum up, I find the study to be clearly presented and well prepared.

 Additional notes:

- To my point of view, the data were collected competently to reach higher multiplicity (which is indispensable for SAD phasing) for P1 space group by exploiting kappa goniometer.
- The workflow for structure solution via Cd-SAD presented in this article seems to be relatively straightforward even considering the problem with centrosymmetric Cd sites (easily resolved by chain tracing).
- "The phasing of protein crystals by SAD starts from finding the positions of an anomalous substructure, which is usually done by direct methods." - In fact, the process involves Patterson search coupled with direct methods-based calculations ^1^ namely dual-space refinement.
- I might suggest adding a few words on how the authors introduced cadmium ions into the crystal (I see that it was co-crystallisation as reported in their previous article).

I have read this submission. I believe that I have an appropriate level of expertise to confirm that it is of an acceptable scientific standard.

## References

[1] SchneiderTSheldrickG: Substructure solution withSHELXD. *Acta Crystallographica Section D Biological Crystallography*.2002;58(10) : 10.1107/S0907444902011678 1772-1779 10.1107/S0907444902011678 12351820

# Peer review: Reviewer response for version 1

**DOI:** 10.5256/f1000research.19346.r43346

**Published:** 2019-02-18

**Authors:** Thomas E. Edwards

**Affiliations:** 1 Bainbridge Island, Bainbridge Island, WA, 98110, USA

Eliseev et al. demonstrate the ability to use cadmium divalent cations, X-ray data collected on an in house X-ray source (Cu Kalpha), and single wavelength anomalous dispersion (SAD) phasing to determine the structure of a protein, in this case the anti-ErbB3 single domain antibody (sdAb). They originally solved the structure by molecular replacement (MR) and reported it in a previous publication, but here they demonstrate the applicability of Cd-SAD to this data set to determine the structure. By our examination of the literature, at least two other structures have been solved by Cd-SAD but both of these structures were solved using data collected at a synchrotron source at a longer wavelength (PDB IDs 2X7K and 5AM6). This particular application provides an excellent alternative method to others presented in the literature and referenced in this paper.

 A couple of notes and comments:

1. The authors may help the reader understand the number of electron possible for phasing by referring the reader to the UW X-ray anomalous scattering web page (http://skuld.bmsc.washington.edu/scatter/) designed by Ethan Merritt.
2. The authors do an excellent job of data collection on their in house instrument by using the kappa goniometer to increase completeness and enhance the anomalous signal.
3. The authors should also site a second paper by Terwilliger et al. from the same issue as reference 15 but with pages 359-374 ^1^ which provides additional information on experimental design and execution.
4. We performed an overnight soak at 5 mM CdCl2, collected a high resolution data set in house on a Cu Kalpha source, and obtained sufficient anomalous signal to solve the structure by Cd-SAD. This provided us with good confidence that this method should be generally applicable. 

Overall, the current paper by Eliseev provides a good alternative approach to experimental structure determination, complementing previously reported techniques.

I have read this submission. I believe that I have an appropriate level of expertise to confirm that it is of an acceptable scientific standard.

## References

[1] TerwilligerTCBunkócziGHungLWZwartPHSmithJLAkeyDLAdamsPD: Can I solve my structure by SAD phasing? Planning an experiment, scaling data and evaluating the useful anomalous correlation and anomalous signal. *Acta Crystallogr D Struct Biol*.2016;72(Pt 3) : 10.1107/S2059798315019403 359-74 10.1107/S2059798315019403 26960123PMC4784667
